# Advances in the understanding of mitochondrial DNA as a pathogenic factor in inflammatory diseases

**DOI:** 10.12688/f1000research.10397.1

**Published:** 2017-02-20

**Authors:** Ray K. Boyapati, Arina Tamborska, David A. Dorward, Gwo-Tzer Ho

**Affiliations:** 1 MRC Centre for Inflammation Research Queens Medical Research Institute, University of Edinburgh, 47 Little France Crescent, Edinburgh, EH16 4TJ, UK; 2 Department of Gastroenterology, Monash Health, Clayton, VIC, Australia

**Keywords:** mitochondrial DNA, mtDNA, mtDNA-mediated inflammation, inflammatory diseases

## Abstract

Mitochondrial DNA (mtDNA) has many similarities with bacterial DNA because of their shared common ancestry. Increasing evidence demonstrates mtDNA to be a potent danger signal that is recognised by the innate immune system and can directly modulate the inflammatory response. In humans, elevated circulating mtDNA is found in conditions with significant tissue injury such as trauma and sepsis and increasingly in chronic organ-specific and systemic illnesses such as steatohepatitis and systemic lupus erythematosus. In this review, we examine our current understanding of mtDNA-mediated inflammation and how the mechanisms regulating mitochondrial homeostasis and mtDNA release represent exciting and previously under-recognised important factors in many human inflammatory diseases, offering many new translational opportunities.

## Introduction

Mitochondria are intracellular double-membrane-bound organelles (“cellular powerhouses”) with many essential physiological roles in energy production, programmed cell death, calcium homeostasis, and the synthesis of lipids, amino acids, and haem. In addition, they are involved in antibacterial, antiviral, and stress responses to hypoxia and tissue injury ^1, 2^. Mitochondria are evolutionarily derived from energy-producing alpha-bacteria, engulfed by archezoan cells approximately 2 billion years ago leading to a symbiotic relationship that forms the basis of the eukaryotic cells ^3^. The mitochondria share several features with bacteria, including the double-membrane structure, a circular genome that replicates independently of nuclear DNA, and the synthesis of *N*-formylated proteins ^4^. As the innate immune system recognises conserved bacterial molecules, mitochondrial constituents are similarly immunogenic, acting as damage-associated molecular patterns (DAMPs) when released into the cytosol and extracellular environment, triggering innate immune responses, and promoting inflammation ^5^. In this review, we focus particularly on the role of mitochondrial DNA (mtDNA) as a specific inflammatory factor, the mechanisms behind its abnormal release, and its effects on downstream inflammatory pathways in human inflammatory diseases.

## Elevated circulating mtDNA in human diseases

**Figure 1.**
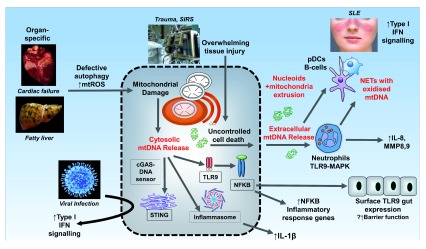
The contribution of mitochondrial DNA to disease pathogenesis. Medical conditions are in italics. Where and how mitochondria are released are indicated in red. Box in dotted line frames mitochondrial DNA (mtDNA) sensor target. cGAS, cyclic GMP-AMP synthetase; IFN, interferon; IL, interleukin; MAPK, mitogen-activated protein kinase; MMP, matrix metalloproteinase; mtROS, mitochondria-derived reactive oxygen species; NET, neutrophil extracellular trap; NFκB, nuclear factor kappa B; pDC, plasmacytoid dendritic cell; SIRS, systemic inflammatory response syndrome; SLE, systemic lupus erythematosus; STING, stimulator of interferon genes; TLR9, Toll-like receptor 9.

Freely circulating mtDNA can be detected, and over 60 studies have quantified mtDNA by quantitative polymerase chain reaction (PCR) in plasma and serum in human diseases ( Table 1). In general, they are increased in conditions with acute tissue injury such as trauma, acute myocardial infarction, and sepsis, implicating major cellular stress and uncontrolled cell death as key factors in the release of mtDNA ( Figure 1). In cancer, where its role as “liquid biopsies” is a topic of considerable interest, the pattern is less clear, and relatively lower circulating levels are found in some cancers ^6^.

**Table 1.** Circulating mitochondrial DNA in human disease.

Disease category	Disease	Blood fraction	Finding	Reference(s)
Trauma	Trauma	Plasma	High mtDNA levels in trauma compared with HCs and correlated with injury severity	7
	Trauma	Plasma	High mtDNA levels in trauma	8, 9
	Trauma with MODS	Plasma	Higher levels of mtDNA had higher relative risk for mortality Higher levels of mtDNA in those with SIRS/MODS compared with those without	10
	Trauma and severe sepsis	Plasma	mtDNA higher in patients with trauma compared with HCs on day 1 mtDNA correlates with injury severity scores in trauma patients mtDNA higher on day 1 in non-survivors compared with survivors	11
	Post-traumatic SIRS	Plasma	mtDNA is an independent predictor for post-traumatic SIRS	12
	Trauma	Plasma	mtDNA higher in trauma patients with correlation with injury severity	7
	Trauma (femur fracture)	Plasma	mtDNA higher in trauma patients than HCs	13
	Trauma	Plasma	mtDNA higher in trauma patients compared with HCs at two time points (pre-hospital and day 1)	14
	Trauma	Plasma	mtDNA higher in trauma patients than HCs mtDNA higher in non-survivors compared with survivors	15
Sepsis	Severe sepsis	Plasma	mtDNA higher in patients with severe sepsis compared with HCs No significant difference in mtDNA between non-survivors and survivors in severe sepsis	11
	Severe sepsis in the ED	Plasma	mtDNA higher on admission in severe septic patients than in HCs mtDNA is higher in non-survivors than in survivors, increases initially and gradually decreases after antimicrobial therapy, and is an independent predictor of fatality	16
	Sepsis	Plasma	mtDNA higher in septic patients compared with HCs	17
	Septic shock	Plasma	mtDNA higher in patients with septic shock	18
	Adult community- acquired bacterial meningitis	Plasma	mtDNA levels were higher in patients with aseptic or bacterial meningitis compared with HCs mtDNA levels fall during course of admission High mtDNA levels associated with poorer outcome in adult community-acquired bacterial meningitis	19
	Infectious SIRS	Plasma	mtDNA higher in septic patients compared with HCs	20
	Paediatric sepsis	Plasma	mtDNA higher in septic patients compared with critically ill non- septic and HC patients	21
	Severe sepsis in the ED	Plasma	No significant difference in mtDNA between sepsis and HC cohorts	22
Critically ill patients	ICU patients	Plasma	Increased mtDNA levels associated with medical ICU mortality	23
	Critically ill patients (in the ICU)	Plasma	Patients with highest quartile of mtDNA in plasma had higher risk of dying When stratified by TLR9 expression, only patients with high expression of TLR9 had an association with mortality and mtDNA level	24
	Out-of-hospital cardiac arrest	Plasma	Significantly higher levels in non-survivors than in survivors	56 ^a^
Liver failure	Acetaminophen- induced acute liver failure	Serum	mtDNA higher in acetaminophen-induced acute liver failure patients compared with HCs mtDNA higher in non-survivors compared with survivors	25
	Acetaminophen- induced acute liver injury	Plasma	mtDNA higher in patients with acetaminophen overdose with abnormal liver function tests compared with HCs and those with acetaminophen overdose but normal liver function tests	27
	Fulminant liver failure	Serum	Higher during acute liver injury	26
Heart disease	AMI	Plasma	Significantly higher mtDNA in ST elevation myocardial infarction patients than in stable angina pectoris patients (reducing rapidly to similar levels 3 days after PCI)	28
	AMI	Plasma	Significantly higher levels in AMI patients compared with HCs Levels dropped to normal immediately after PCI	29
	AMI	Plasma	Significantly higher levels in acute AMI patients compared with HCs on admission	30
	T2DM with CAD	Plasma	Significantly elevated levels in T2DM compared with HCs Higher levels in those with diabetes mellitus and CAD compared with those without CAD mtDNA levels correlated with C-reactive protein in patients with CAD	31
	T2DM with CAD	Plasma	Significantly higher levels in CAD patients with T2DM	32
	Heart failure	Plasma	Higher levels of mtDNA in heart failure patients compared with age- and sex-matched HCs; no association with disease severity	110
Stroke	Acute ischaemic stroke	Plasma	mtDNA levels higher in acute cerebral infarction than in HCs No significant difference in mtDNA between good versus poor outcome cohorts	33
	Subarachnoid haemorrhage	Plasma	No significant difference in mtDNA between subarachnoid haemorrahge and HC groups Overall plasma mtDNA not a good marker of prognosis	34
	Intracerebral haemorrhage	Plasma	No significant difference in mtDNA between intracerebral haemorrhage and HC groups No correlation between mtDNA and disease severity	35
Malignancy	Breast cancer	Plasma	Reduced levels of mtDNA in benign or malignant breast cancer compared with HCs	111
	Ovarian cancer	Plasma and serum	Plasma: significantly higher levels of mtDNA in ovarian cancer group compared with HCs and ovarian benign tumour group Serum: no significant difference between groups above	112
	Testicular germ cell cancer	Serum	mtDNA levels were significantly higher in patients with testicular cancer than in HCs, although it did not correlate with any clinicopathological variable of disease status	113
	Urological malignancies	Serum	mtDNA were significantly higher in “urological malignancies” (bladder cell, renal cell, and prostate cancer)	114
	Prostate cancer	Serum	mtDNA could not distinguish between benign prostatic hypertrophy and prostate cancer Patients with early biochemical recurrence after radical prostatectomy have higher mtDNA levels	115
	Ewing’s sarcoma	Serum	mtDNA significantly lower in patients with Ewing’s sarcoma compared with HCs	116
	Lung cancer	Serum	mtDNA significantly higher in lung cancer patients compared with those with benign lung diseases and healthy individuals and closely associated with tumour, lymph node, metastasis (TNM) stage	117
	Advanced prostate cancer	Plasma	mtDNA levels are elevated in advanced prostate cancer patients and are associated with decreased survival	118
	Adenocarcinoma of the lung in patients receiving erlotinib	Plasma	Rise in mtDNA levels in patients with partial response; drop in mtDNA levels in those with progressive disease or no response No correlation with progression-free survival	119
	Exposure to carcinogenic halo-alkane-based pesticides	Serum	Exposure to these carcinogens was significantly associated with elevated serum levels of circulating mtDNA (case control study)	120
	Renal cell carcinoma	Plasma	Higher levels in metastatic compared with non-metastatic patients and controls	121
HIV	HIV	Plasma	Higher levels in acute HIV infection, late presenters compared with long-term non-progressors and HCs Also correlated with viral load	122
	Lipodystrophy in HIV patients treated with highly active anti-retroviral therapy	Plasma	Significantly higher levels in HIV-infected versus non-infected individuals Significantly higher levels in those with lipodystrophy compared with those without lipodystrophy at month 24	123
	HIV	Plasma	No significant association between HIV disease status and mtDNA	124
Inflammatory autoimmune conditions	Rheumatoid arthritis	Plasma	Higher percentage of detectable levels in rheumatoid arthritis patients compared with controls	37 ^b^
	Granulomatosis with polyangiitis	Serum	Significantly higher levels in granulomatosis with polyangiitis patients compared with controls	38
Age and exercise	Age	Plasma	mtDNA levels increased gradually after the fifth decade of life	125
	Age	Plasma	No association with age but mtDNA associated with HLA-DR	126
	Aging and “frailty”	Plasma	Aging: no difference in mtDNA between younger and older subjects Frailty: mtDNA copy number directly correlated with frailty score	127
	Exercise	Plasma	Reduced mtDNA in response to exercise	128
	Male volleyball players	Plasma	Lower levels in professional volleyball players compared with healthy non-athlete controls	129
Miscellaneous	Corrosive injury (gastrointestinal ingestion)	Plasma	Significantly higher mtDNA in mortality group versus survival group at presentation and after 12 hours	130
	Pulmonary embolism	Plasma	Predictor of 15-day mortality	131 ^c^
	Autism	Serum	Significantly higher mtDNA in young autistic children compared with HCs	132
	Haemodialysis	Plasma	Significantly higher levels in maintenance haemodialysis patients compared with HCs	133
	End-stage renal failure in Han population	Plasma	End-stage renal failure patients had higher mtDNA copy number	134
	Bipolar disorder	Serum	No difference between bipolar disorder and HC groups Higher levels in bipolar disorder group compared with sepsis	135
	Low levels of ionising radiation	Serum	Higher levels in interventional cardiologists exposed to low levels of ionising radiation compared with controls	136
	Friedreich’s ataxia	Plasma	Significantly reduced mtDNA in Friedreich’s ataxia patients compared with HCs	137
	Non-haemolytic transfusion reaction	Platelet concentrates	Higher mtDNA copy number in non-haemolytic transfusion reaction platelet concentrate versus normal platelet concentrate	138

This table lists studies reporting mitochondrial DNA (mtDNA) analysed by polymerase chain reaction (PCR) on serum or plasma—that is, circulating as a damage-associated molecular pattern (DAMP)—in human diseases. ^a^Letter. ^b^PCR rather than quantitative PCR used. ^c^Earlier study in 2010 not included. AMI, acute myocardial infarction; CAD, coronary artery disease; ED, emergency department; HC, healthy control; HIV, human immunodeficiency virus; HLA-DR, human leukocyte antigen–antigen D related; ICU, intensive care unit; MODS, multiple organ dysfunction syndrome; PCI, percutaneous coronary intervention; SIRS, systemic inflammatory response syndrome; T2DM, type 2 diabetes mellitus; TLR9, Toll-like receptor 9.

### Systemic inflammatory response syndrome

Systemic inflammatory response syndrome (SIRS) is a serious condition associated with high mortality, and affected individuals display progressive signs or symptoms of systemic upset reflecting widespread inflammation, often involving multiple organ dysfunction and failure (for example, lungs, kidneys, and brain). SIRS is often a result of major sepsis but also commonly occurs in the context of injury such as trauma. An early study by Lam *et al.* found that individuals admitted for blunt traumatic injury had increased plasma nuclear DNA and mtDNA levels ^7^. Subsequently, Hauser *et al*. made the seminal observation that it is the freely circulating mtDNA following traumatic injury which possesses the distinct ability to trigger and drive the clinical manifestation of SIRS ^8^. Several studies have confirmed the observation of elevated plasma mtDNA in trauma and SIRS ^9– 15^. A number of studies have found correlations with injury severity in trauma ^7, 11^ and higher mtDNA in non-survivors compared with survivors ^11, 15^. Furthermore, Gu *et al*. found that elevated plasma mtDNA was an independent predictor of SIRS in trauma patients ^12^. In sepsis, elevated levels of circulating mtDNA have also been found in multiple studies ^11, 16– 21^. De Caro *et al*. found higher mtDNA in the plasma of critically ill paediatric patients who were septic compared with similarly unwell but non-septic patients ^21^. ** The one negative study in sepsis may be explained by numerous factors, including a relatively well patient cohort, only one “spot” measurement being taken at presentation, and the potentially confounding factor of cellular content/debris ^22^. Studies of patients in the intensive care setting have found that higher mtDNA levels are associated with poorer outcomes ^23, 24^.

### Acute single-organ injury: liver, heart, and brain

High levels of mtDNA are present in the serum and plasma of patients with acute injury to a variety of single organs. Acetaminophen overdose induces massive hepatocyte necrosis and in severe cases can lead to multi-organ failure and remains one of the commonest indications for liver transplantation. In fulminant liver failure secondary to acetaminophen overdose, mtDNA in the serum was found to be 30 to 40 times higher than normal, and non-survivors had higher levels than survivors ^25^; a separate study of drug-induced acute liver failure found serum mtDNA levels to be 10,000-fold higher ^26^. Serum mtDNA of acetaminophen overdose patients with derangement in the liver enzyme alanine aminotransferase (a marker of hepatocyte damage) is significantly higher than that of overdose patients who had normal liver enzymes ^27^, suggesting that the extent of mtDNA release into the circulation depends on the extent of hepatocyte necrosis. Similarly, extensive cardiomyocyte necrosis is found in acute myocardial infarction, which is also associated with elevated mtDNA in multiple studies ^28– 30^ and falls after angioplasty or coronary stent insertion to restore blood flow to the damaged myocardium ^28, 29^. Patients with diabetes mellitus and coronary artery disease have higher mtDNA levels than those with diabetes but without coronary artery disease ^31, 32^. mtDNA is also higher in acute cerebral ischaemia, caused by a reduction in cerebral blood flow by embolus or local thrombosis, and plasma levels gradually drop over time after the initial tissue injury ^33^. Interestingly, studies by the same group relating to plasma mtDNA in subarachnoid haemorrhage and spontaneous intracerebral haemorrhage found no significant difference compared with healthy controls, although both were small studies ^34, 35^. Higher mtDNA is found in the cerebrospinal fluid of patients with subarachnoid haemorrhage ^34^ and traumatic brain injury ^36^ and is associated with worse clinical outcomes. Overall, in these conditions, significant mtDNA release following massive tissue or cellular injury is evident and likely contributes to the uncontrolled inflammatory response ^25^.

### Chronic inflammatory and immune-mediated diseases

The role for mtDNA in immune-mediated inflammatory diseases, unlike conditions relating to injury, is now also emerging. In rheumatoid arthritis, a chronic relapsing autoimmune condition affecting the joints, mtDNA was present in the plasma and synovial fluid of most patients but undetectable in healthy controls ^37^. Similarly, higher plasma mtDNA is found in granulomatosis with polyangiitis, an autoimmune disease whose features include necrotising granulomatous inflammation and vasculitis ^38^. Systemic lupus erythematosus (SLE) is a multi-organ autoimmune disease with hallmarks including excessive type I interferon (IFN) and antibodies against nucleic acids. Caielli *et al*. explored the potential pathogenic importance of oxidised mtDNA in SLE ^39^. They showed that there is a defect in mitochondrial clearance that leads to abnormal extrusion of oxidised mtDNA, which triggers a subsequent interferogenic response. Elevated anti-mtDNA antibodies were found in a separate study of SLE, particularly in lupus nephritis, where levels correlated with the lupus nephritis activity index better than anti-double-stranded DNA (anti-dsDNA) antibody levels did ^40^. In a further study of SLE, neutrophil extracellular traps (NETs) released from the inflammatory subset of low-density granulocyte were highly enriched in mtDNA compared with NETs from healthy control neutrophils ^41^. NETs are networks of extracellular fibres that are primarily composed of DNA and that are strikingly expelled following a form of neutrophil cell death (NETosis) with an aim to control pathogens; however, this study demonstrates that mtDNA-enriched NETs are pro-inflammatory in nature. Similar findings are reported in chronic granulomatous disease in this study. Higher levels of mtDNA have been found in the chronic inflammatory states of HIV (although not in all studies), end-stage renal failure, and diabetes mellitus ( Table 1). In obese individuals with steatohepatitis, mitochondria enclosed in microparticles can also be detected in plasma ^42^. These findings suggest that mtDNA, otherwise a “self-signal”, may be an active component in the aberrant immune or inflammatory response in chronic diseases and in autoimmunity.

## mtDNA contributes to inflammatory response

mtDNA was first directly implicated as a key factor in the development of inflammatory pathology over a decade ago when intra-articular injection of oxidised mtDNA, but not nuclear DNA, triggered inflammatory arthritis in mice ^43^. There are now numerous studies using *in vivo* injection of mtDNA to provoke local or systemic inflammation or both ^9, 44– 46^. Moreover, there are now several *in vivo* studies to show that genetic deletion or pharmacologic interference of these pathways reduces the inflammatory effect of mtDNA (as will be discussed in the next section). Hence, it is clear that mtDNA release is not an epiphenomenon but directly contributes to the genesis of inflammation ( Figure 1). Current evidence shows that mtDNA-mediated inflammation is predominantly driven by the Toll-like receptor 9 (TLR9), inflammasome, and, more recently, stimulator of interferon genes (STING) pathways.

### Toll-like receptor 9

TLR9 is located in the endoplasmic reticulum (ER) of various immune cells and translocates to the endosome upon sensing of hypomethylated DNA with CpG motifs, such as bacterial DNA ^47, 48^. Given its high frequency of unmethylated CpG dinucleotide repeats, it is postulated that mtDNA mediates inflammation dependent on the TLR9 pathway and potentially exerts a similar effect as on bacterial CpG. TLR9 recognises a variety of types of oligodeoxynucleotides (ODNs); for example, class A ODNs preferentially activate plasmacytoid dendritic cells whilst class B CpG ODNs activate B cells ^49^. Some of our understanding of how mtDNA may interact with TLR9 is extrapolated from work with class A ODNs, although they do not necessarily have the same effect. After activation of TLR9 by CpG DNA, inflammatory cytokine induction and Th1 immune responses occur ^50^ and TLR9 is necessary in CpG DNA-driven responses ^51^. TLR9 ligands can preferentially activate downstream pathways, including pro-inflammatory nuclear factor kappa B (NFκB), nucleotide-bindingdomain and leucine-rich repeat (NLR) pyrin domain containing 3 (NLRP3) inflammasomes, and interferon regulatory factor (IRF)-dependent type 1 IFN, which can upregulate IL-1 receptor antagonist ^52, 53^.

Most tissue injury models show better outcomes when the *tlr9* gene is deleted. Wei *et al*. recently observed that *tlr9 ^−/−^* mice have improved survival outcome in a necrotic lung model of cationic nanocarrier-induced necrosis and mtDNA release *in vivo* ^54^. Furthermore, the pulmonary inflammation seen after injection of mtDNA was significantly reduced in *tlr9 ^−/−^* and *MyD88 ^−/−^* mice, underlining the importance of the TLR9–MyD88 pathway ^54^. Intravenous injection of mitochondrial debris with substantial amounts of mtDNA into mice induced a systemic inflammatory response in wild-type mice that was significantly attenuated in *tlr9 ^−/−^* mice ^45^. *Tlr9 ^−/−^* mice also have better survival compared with wild-type counterparts in severe renal ischaemia reperfusion injury with associated decreased circulating mtDNA ^55^. A similar protective effect is seen in *tlr9 ^−/−^* mice with acute acetaminophen overdose with observed lower serum mtDNA and an absence of lung inflammation in contrast to the findings of wild-type mice ^26^. Nevertheless, the reduction in mtDNA in *tlr9 ^−/−^* mice is intriguing and could be explained by the reduced inflammation with lower resultant cellular necrosis. Alternatively, it is possible that TLR9 is somehow involved in mtDNA release into the extracellular circulation. In a recent study using a murine model of non-alcoholic steatohepatitis (NASH), mtDNA from NASH hepatocytes resulted in greater activation of TLR9 than did mtDNA from control livers ^42^. This suggests that mtDNA that is selectively modified during pathologic disease processes can augment the ensuing inflammatory response. Similarly, the level of TLR9 expression (due to various factors) appears to be important. In those with high mtDNA levels, higher TLR9 expression is associated with increased mortality in the intensive care unit (ICU), as discussed earlier ^56^.

Neutrophils have received the most attention in studies on mtDNA–TLR9 signalling in several different inflammatory settings. Zhang *et al*. found that mtDNA activates neutrophil p38 mitogen-activated protein kinase (MAPK) through TLR9 with release of matrix metalloproteinase 8 (MMP8) and MMP9 ^8, 9^, a finding confirmed in a study in which phosphorylated p38 and MMP9 increased after mtDNA treatment of neutrophils ^57^. A separate study reported similar findings where pre-treatment with TLR9 inhibitor ODN2088 inhibited the activation of p38 MAPK and release of MMP8 ^54^. Gu *et al*. also found that intratracheal administration of mtDNA provokes lung inflammation through TLR9–p38 MAPK ^58^. Hip fracture in rats resulted in mtDNA release into the circulation as well as higher TLR9 and NFκB p65 activation and subsequent lung injury ^46^. The role of other MAPKs such as extracellular signal-regulated kinases (ERKs) and c-Jun N-terminal kinases (JNKs) remains unclear and, to our knowledge, unexamined in this context. These data suggest a pathway where mtDNA activates neutrophils through TLR9 binding and activation of the MAPK pathway with subsequent MMP8 and MMP9 release ( Figure 1).

When mtDNA is considered vis-à-vis the site and location of TLR9 receptor, mtDNA must be either displaced from whole mitochondria and moved into the cytosol or, when extracellular, internalised by some mechanism(s) to act on endosomal TLR9. The endosomal location of TLR9 is most likely a mechanism to avoid unwanted activation ^59^. It is unclear how extracellular mtDNA is internalised, but possibilities include endocytosis, transmembrane diffusion, phagocytosis, and receptor-mediated endocytosis ^60^. Transmembrane diffusion is unlikely because of the highly (negatively) charged nature of DNA, which makes it difficult to pass through the cellular membrane. A recent study found that monocyte-derived macrophages can take up whole mitochondria released from necroptosis, suggesting that phagocytosis could be a relevant mechanism ^61^. The macrophage has a clear role in resolving inflammation by clearing up cellular debris and apoptotic bodies by phagocytosis. When mitochondria are not cleared during non-apoptotic cell death, the macrophage may phagocytose cellular corpses with mtDNA still abundantly present. Typically, apoptotic corpses can suppress the transcription of pro-inflammatory cytokine genes, promote the secretion of anti-inflammatory cytokines by phagocytes, and cause antigen-presenting cells to present dead cell antigen in a manner that promotes immunological tolerance (reviewed by Zitvogel *et al.* ^62^) *.* It will be of interest to consider the fate of mtDNA when macrophages or dendritic cells phagocytose cellular corpses with mtDNA. Does this clear the mtDNA or does it regulate subsequent functions (for example, immune responsiveness) in these cell types? This has yet to be studied in detail. It is also possible that binding to additional cofactors facilitates the internalisation into immune cells, and, in this instance, high-mobility group box 1 (HMGB1) and receptor for advanced glycation end products (RAGE) have been implicated ^63^. In this study, HMGB1–CpG (class A) complexes resulted in TLR9/RAGE association and recruitment of MyD88 in B cells ^63^. Here, RAGE was visualised as associating with the DNA and was internalised with some sequestered in endosome-like structures. However, this possible mechanism requires further investigation. It has also been proposed that activation of autoreactive B cells by CpG DNA occurs after B-cell receptor engagement, leading to the delivery of CpG DNA to endosomal TLR9 ^64^.

Although nucleic acid-sensing TLRs on immune cells are found mainly within cells, cell surface expression has also been described. Via flow cytometry, TLR9 has been detected on the surface of resting B lymphocytes ^65, 66^ and peripheral blood mononuclear cells ^67, 68^. One functional *ex vivo* study found primary human and mouse TLR9 surface expression in neutrophils, which are upregulated by a variety of stimuli, including TLR9 agonists ^69^. However, it remains unclear whether TLR9 is able to signal from the cell surface. In other cell types, TLR9 is also expressed on the cell surface. For example, TLR9 is expressed on both the apical and the basolateral membranes of intestinal epithelial cells, although NFκB is activated only via basolateral stimulation of CpG ligands ^70, 71^. This is relevant at the gut mucosal interface, as this limits the extent of TLR9 activation at the apical surface, which is in contact with a luminal milieu rich with bacterial DNA. Hence, compromised intestinal barrier integrity and translocation of bacterial CpG from the lumen during gut pathology will lead to basolateral stimulation in this context. Whether mtDNA has a different propensity compared with bacterial CpG to trigger TLR9 depending on epithelial site has not been studied.

### The inflammasome

The inflammasomes are targets of mtDNA leading to cleavage and activation of caspase-1 and downstream maturation of interleukin-1β (IL-1β) and IL-18 ^72^. Here, it is the cytosolic release of mtDNA that exerts the dominant effect on inflammasome activation. Of the several inflammasomes described, the NLRP3 inflammasome is the best characterised in this regard. Nakahira *et al*. showed that depletion of mtDNA reduced IL-1β secretion in macrophages following treatment with known inflammasome triggers lipopolysaccharide (LPS) and ATP ^73^. Of interest, mitochondria-derived reactive oxygen species (mtROS) is a further key mediator in this process. Pharmacologic induction of mtROS correlates with higher secretion of active IL-1β in an NLRP3- and caspase-1-dependent manner, and treatment with mtROS scavengers suppresses this effect ^74^. The requirement for mtROS in NLRP3 activation has been confirmed by other studies ^73, 75– 77^ and may be explained by its oxidising effects on mtDNA. Shimada *et al*. showed that it is the oxidised form of mtDNA that confers the inflammatogenic potential to mtDNA ^75^. mtROS enhances not only the oxidative process but also the cytosolic translocation of oxidised mtDNA that then binds directly to NLRP3 ^75^. Non-oxidised mtDNA is insufficient to activate the NLRP3 inflammasome, although it may stimulate IL-1β production via other inflammasomes such as the absent in melanoma 2 (AIM2) ^78^. Interestingly, genetic deletion of NLRP3 and caspase-1 results in less mtDNA release ^73, 77^. This suggests a positive-feedback loop, in which activation of the NLRP3 inflammasome by oxidised mtDNA further promotes mtDNA release. The overwhelming or persisting (or both) ROS production by inflammatory cells, for example, is known to damage macromolecules (DNA as well as RNA, lipids, carbohydrates, and proteins) of the surrounding cells. Activated neutrophils produce large amounts of ROS as part of their essential role in host defense ^79^. Hence, this is a likely major contributory factor to mtDNA damage once the inflammatory process is triggered.

Other factors controlling mitochondria-mediated NLRP3 activation are also relevant. For example, defective autophagy increases caspase-1 activation, IL-1β and IL-18 production, and cytosolic mtDNA translocation in LPS- and ATP-primed macrophages ^76^. Pharmacological inhibition of mitophagy/autophagy in human macrophages results in the accumulation of damaged mitochondria, ROS generation and IL-1β secretion ^74^, and increased NLRP3 expression in the presence of LPS ^80^. Hence, defective autophagy leads to inadequate clearance of damaged mitochondria, priming the internal cellular environment for NLRP3 activation. It is noteworthy that, given the diversity of NLRP3 activators, current literature suggests that the precise mechanism of NLRP3 activation is still under debate ^81^. Although the role of the inflammasome is often considered separately from TLR9 here, there is evidence that TLR/NFκB activation is a necessary priming step leading to NLRP3 upregulation and subsequent downstream signalling. NFκB-activating stimulus is required for cells to express pro-IL-1β and NLRP3 ^82^. Imeada *et al*. showed that stimulation of TLR9 by DNA fragments during early acetaminophen-induced cell death can lead to the transcriptional activation of the IL-1β gene, resulting in the formation of pro-IL-1β ^83^. Using the acetaminophen hepatotoxicity model, they showed that *NLRP3* deletion and related inflammasome components *ASC* and *Caspase-1* were protective against induced liver failure ^83^. A further study, however, did not show any effect of NLRP3 deletion on the outcomes of acetaminophen-induced liver failure ^84^. Hence, in the context of liver necrosis, the role for NLRP3 inflammasome remains controversial.

### STING pathway

The role of mtDNA in innate immunity through the STING pathway has also been a focus of recent studies. STING is a cytosolic protein anchored to the ER ^85^. STING can be activated either by direct association with dsDNA or by cyclic dinucleotides, which can be derived from intracellular bacteria or viruses or produced by a DNA sensor, cyclic GMP–AMP (cGAMP) synthetase (cGAS) ^86^. This, in turn, activates IRF3, which ultimately translocates to the nucleus and transcribes type I IFN genes, and also the NFκB pathway ^85^.

Two independent groups recently discovered that the STING-mediated IFN response can also be activated by mtDNA ^87, 88^. They first observed that deficiency of apoptotic caspases (3, 7, and 9) resulted in upregulation of type I IFN genes. This response was dependent on Bak/Bax, pro-apoptotic proteins responsible for mitochondrial outer membrane permeabilisation leading to mtDNA release, and the release of cytochrome C, which activates the intrinsic apoptotic pathway. Typically, apoptosis is considered immunologic-silent; for example, it does not trigger an inflammatory response. However, these studies demonstrated that, when caspases (9 and 3/7) responsible for the completion of apoptotic process are inhibited or deleted, cytosolic mtDNA goes on to activate cGAS/STING-mediated type I IFN signalling ^87, 88^. Hence, these caspases serve as a “brake” on the mtDNA-inflammatory effect during cell death. mtDNA released during cell death has been previously reported to provide a second signal that cooperates with an additional inflammatory signal (for example, LPS) to activate the NLRP3 inflammasome and induce IL-1β production in murine macrophages ^75^. Further evidence of an mtDNA role in STING-mediated IFN responses comes from West *et al*. ^89^. Here, partial deficiency of the mtDNA-binding protein mitochondrial transcription factor A (TFAM) was associated with increased concentrations of cytosolic mtDNA and enhanced type I IFN response, which was attenuated by knockdown of components of the STING pathway.

Aberrant mtDNA–STING signalling has also been implicated in human inflammatory diseases, such as SLE. As discussed earlier, Lood *et al*. showed that treatment of human neutrophils with SLE-abundant ribonucleoprotein immune complexes induces mtROS, mtDNA oxidation, and translocation of the mitochondria to the plasma membrane ^41^. Oxidised mtDNA is then released extracellularly as a component of NETs. Transfection of NET-derived mtDNA results in expression of IFN-β in human peripheral mononuclear cells. Systemic injection of oxidised mtDNA increases IFN-stimulated gene expression in the spleen of wild-type but not STING-deficient mice. Similar to inflammasomes, uncontrolled mtROS production promoting cytosolic mtDNA release is important in STING activation and potentially in the case of autoimmunity. These studies highlight the importance of the innate cellular functions to handle mtDNA release during the initiation of cell death, which ultimately will decide whether the ensuing fate will be that of a silent or inflammatory outcome.

### Mechanisms for mtDNA release

Two levels of mtDNA release—cytosolic and then extracellular—are critically important steps ( Figure 1). In the former, the mechanism of release of mtDNA from mitochondria relies on the opening of mitochondrial permeability transition (MPT) pores in the inner mitochondrial membrane ^90^. Inhibition of pore opening with cyclosporine A resulted in lower mtDNA in the cytosol after stimulation with LPS and ATP ^73^. Ding *et al*. showed that the induction of ROS using oxidised low-density lipoprotein (ox-LDL) increased mtDNA leakage into the cytosol in a dose-dependent manner, and this effect was ameliorated with blockade of the ox-LDL receptor or a ROS inhibitor ^91^.

In terms of extracellular release, cellular stress and necrosis are primary factors in the non-discriminant liberation of a host of mitochondrial components such as mtDNA, N-formyl peptides, ATP, TFAM, and mitochondrial lipids. These mitochondrial constituents also exert their respective effects, which are wide-ranging, on key inflammatory pathways (extensively reviewed by Nakahira *et al*. ^81^). Aside from this non-selective release after uncontrolled cell death, several studies have suggested additional mechanisms such as necroptosis (or programmed necrosis) ^92^. Blood transfusion-induced endothelial necroptosis was recently found to increase extracellular mtDNA as a potential mechanism to explain transfusion-related lung injury ^93^. A recent study suggested that, during necroptosis, mitochondria were released before plasma membrane rupture and then phagocytosed by monocyte-derived macrophages or dendritic cells, triggering an inflammatory response as shown by cytokine production and cell maturation, respectively ^61^. Thus, ingestion of intact mitochondria may represent a distinct uptake mechanism following necroptosis. In a separate study, platelets were also found to be a source for free extracellular mitochondria release and then to act as an endogenous substrate for bactericidal secreted phospholipase A _2_IIA (sPLA _2_-IIA) leading to mitochondrial membrane hydrolysis, loss of mitochondrial structural integrity, and mtDNA release ^94^. Intriguingly, Xin *et al*. found lower levels of mtROS production when metformin was added to activated platelets, and this was associated with decreased extracellular mtDNA release ^95^. The authors found lower complex I activity of the platelet mitochondrial respiratory chain and suggested this as a mechanism for the observed suppressed mitochondrial dysfunction.

Whether there is an active element in mtDNA release is an interesting point of consideration. Active cellular transfer of mitochondria from stromal cells to rescue stricken lung alveoli cells in acute lung injury has been demonstrated ^96^. Extracellular vesicles are important modes of intercellular communication and comprise exosomes (endosomal) and microvesicles (plasma membrane-derived) and are directed by exocytosis. Both chromosomal DNA ^97, 98^ and mtDNA ^99, 100^ have been observed in extracellular vesicles; it has been suggested that the transfer of altered mtDNA between cells may play a role in Alzheimer’s disease and skeletal muscle diseases ^99, 100^. As described earlier, in patients with NASH, a greater percentage of mitochondria was found inside extracellular microparticles and a higher percentage of microparticles contained mitochondria compared with lean subjects ^42^. Furthermore, subjects with NASH had a higher level of oxidised mtDNA in microparticles. Further clarification is required on the concentration and significance of mtDNA in extracellular vesicles and whether this has different immunostimulatory effects compared with cell-free or surface-bound mtDNA. As previously mentioned, the pro-inflammatory effects of mtDNA are dependent on its oxidisation ^75, 101^. The highly oxidative extracellular milieu at sites of tissue inflammation in patients with chronic inflammatory disease may overwhelm anti-oxidant systems, further potentiating the inflammatory potential of DAMPs such as mtDNA ^5^.

### mtDNA degradation and clearance

Several well-described clearance mechanisms limit the pro-inflammatory nature of mtDNA. Autophagy as discussed earlier is important ^102^. Defective autophagy has been implicated in several chronic inflammatory human diseases, including Crohn’s disease ^103^. A proportion of circulating DNA in the bloodstream appears to cross the kidney barrier and be excreted in the urine ^104^. Indeed, mtDNA has been detected in the urine at elevated levels in patients with progressive acute kidney injury ^105^. This may be due to the inflammatory state associated with this condition, the increased clearance with a disturbed kidney barrier, or both. Another possible mechanism of mtDNA clearance is phagocytosis by macrophages in a manner similar to the ingestion of the structurally similar bacterial DNA ^106^. As described earlier, the outcome of phagocytosis of intact mitochondria may be pro- rather than anti-inflammatory; these divergent effects may also be dependent on the phenotype of the phagocytosing cells (for example, inflammatory versus pro-resolution macrophages/monocytes, neutrophils, and red blood cells) ^61, 93^.

In general, non-host DNA in the circulation is digested in part by circulating nucleases, and mtDNA may be affected by a similar mechanism ^107^. Intracellularly, DNases found in the autophagolysosome play a vital role in degrading mtDNA ^102, 108^. Oka *et al*. showed that cardiac-specific deletion of *DNase II* resulted in mtDNA accumulation in cardiomyocytes and the development of heart failure ^102^. In human umbilical vein endothelial cells, lysosomal DNases protect cells against inflammation from mtDNA damage induced by ox-LDL ^91^. Here, small interfering RNA (siRNA) knockdown of DNase II amplifies the mtDNA–TLR9-mediated inflammatory response ^91^. It is unclear whether nucleases have a similar action on mtDNA in the extracellular space or are relevant in the physiological setting, especially when mtDNA is present in microvesicles or housed within intact mitochondria, which protect against DNase II. Intriguingly, DNase pre-treatment abolished renal mitochondrial injury that was observed after injection of mitochondrial debris (including mtDNA) in mice ^45^. However, the precise role of DNase and its effect on the immunostimulatory effects of mtDNA is likely to be more complex, as illustrated by a recent study which showed that DNase II was required for TLR9 activation by bacterial genomic DNA ^109^.

## Conclusions: translational opportunities for mtDNA-mediated inflammation

mtDNA contributes to inflammation at multiple levels when tissue or cellular homeostasis is perturbed. Damaged mtDNA released into the cytosol has a functional short-range effect on immediate “alarm” systems such as the inflammasome and NFκB. Uncontrolled release of mtDNA into the circulation in conditions with significant tissue injury generates a more systemic effect whilst de-regulation of local mitochondrial homeostatic mechanisms such as autophagy or mtROS detoxification contributes to organ-specific pathology as observed in the heart and liver. Failure of such mechanisms may also give rise to a more wide-ranging consequence (for example, in autoimmune diseases such as SLE).

Our review shows that mtDNA-mediated inflammation is important and relevant to many human inflammatory diseases. However, this remains an underexplored field and more insights will likely emerge in the near future. The current evidence offers a rich realm of translational opportunities to target mtDNA-mediated inflammation. There are many plausible approaches, which include targeting cytosolic mtDNA release (for example, directly at MPT using cyclosporine or by specific mitochondrial anti-oxidant strategies, such as MitoQ10 _10_ to reduce mtROS), augmenting clearance (for example, using autophagy activators or correcting factors leading to impaired autophagy), diverting the cellular response following mitochondrial damage (for example, induction of pro-apoptotic caspases), and reducing the inflammatory potential of mtDNA (for example, DNases to digest NET-bound mtDNA and reducing oxidation of mtDNA).

Given that mtDNA can be measured and used as a biomarker, it offers a unique opportunity to stratify and identify individuals who may benefit from specific therapeutic targeting of downstream inflammation pathways (for example, TLR9, NLRP3, or STING pathways). As discussed earlier, there are numerous studies in sepsis, trauma, and acute single-organ injury that have already demonstrated that individuals with high mtDNA levels and TLR9 expressions have worse prognosis. Therefore, there are clear groups in which stratification is useful. However, a number of challenges exist to its implementation as a biomarker, such as the variation in which mtDNA is measured (for example, serum versus plasma, mtDNA-specific PCR primers) and reported in the literature. Standardisation of these protocols, including the identification of “normal” and “abnormal” ranges, will be important prior to clinical use. Furthermore, many studies have failed to include clinically relevant predictive statistics; further studies reporting such statistics in a variety of inflammatory conditions are required.

In conclusion, multiple lines of data show that innate responses to mtDNA, which is similar to and evolutionarily derived from bacteria, are hard-wired into our biology and drive the development inflammation with pathologic consequences in many diseases.
